# Theoretical Investigation of Single-Atom Catalysts for Hydrogen Evolution Reaction Based on Two-Dimensional Tetragonal Mo_3_C_2_

**DOI:** 10.3390/ma17246134

**Published:** 2024-12-15

**Authors:** Bo Xue, Qingfeng Zeng, Shuyin Yu, Kehe Su

**Affiliations:** 1 School of Physical Science and Technology, Northwestern Polytechnical University, Xi’an 710129, China; 2 MSEA International Institute for Materials Genome, Langfang 065500, China; zengqf@dianyunkeji.com (Q.Z.); yusy@dianyunkeji.com (S.Y.); 3 Particle Cloud Biotechnology (Hangzhou) Co., Ltd., Hangzhou 310018, China; 4 Science and Technology on Thermostructural Composite Materials Laboratory, Northwestern Polytechnical University, Xi’an 710072, China; 5 School of Chemistry and Chemical Engineering, Northwestern Polytechnical University, Xi’an 710129, China

**Keywords:** transition metal carbide, single-atom catalysts, hydrogen evolution reaction, density functional theory

## Abstract

Developing highly efficient and cost-competitive electrocatalysts for the hydrogen evolution reaction (HER), which can be applied to hydrogen production by water splitting, is of great significance in the future of the zero-carbon economy. Here, by means of first-principles calculations, we have scrutinized the HER catalytic capacity of single-atom catalysts (SACs) by embedding transition-metal atoms in the C and Mo vacancies of a tetragonal Mo_3_C_2_ slab, where the transition-metal atoms refer to Ti, V, Cr, Mn, Fe, Co, Ni and Cu. All the Mo_3_C_2_-based SACs exhibit excellent electrical conductivity, which is favorable to charge transfer during HER. An effective descriptor, Gibbs free energy difference (Δ*G*_H*_) of hydrogen adsorption, is adopted to evaluate catalytic ability. Apart from SACs with Cr, Mn and Fe located at C vacancies, all the other SACs can act as excellent catalysts for HER.

## 1. Introduction

As a clean technology to convert renewable energies into electrical and thermal energy, the production of hydrogen from water splitting has drawn a lot of attention for several decades [1,2,3]. During water splitting, the hydrogen evolution reaction (HER) plays a critical role in producing hydrogen from water. The efficiency of HER highly relies on the performance of the catalyst. Currently, the most effective electrocatalysts for HER are Pt and Pt-based materials [4]. Unfortunately, the severe scarcity of Pt on earth leads to its high price and limits the wide-scale industrial application of such catalysts [5]. The development of inexpensive and stable catalysts for HER is crucial for the realization of the hydrogen economy, which is based on hydrogen production and storage. Up to now, considerable effort has been devoted to finding alternatives based on abundant elements with high electrolytic efficiency for HER, such as nanostructured metal carbides [6,7], carbon doped with heteroatoms [8,9,10] and transition metal oxides [11]. However, the catalytic performance of most Pt-free catalysts is overshadowed by Pt-based catalysts. Discovering Pt-free HER catalysts with high efficiency is still a great challenge.

On account of large specific surface area and high catalytic activity, two-dimensional (2D) materials have great potential in HER catalysis [12]. Among the 2D materials, 2D transition metal carbides (TMCs) have been widely studied to investigate their potential as catalysts for HER, and many TMC-based materials were found to have catalytic ability. A class of 2D TMCs known as MXenes has received great interest for their catalytic performance in HER. MXenes have a general formula of M*_n_*_+1_X*_n_*T*_x_* (*n* = 1–3), where M represents the transition metal (such as V, Mo, Ti, Nb and so on), X denotes nitrogen and/or carbon, and T*_x_* stands for surface termination (such as -O, -F, or OH) [13]. Usually, MXenes are synthesized by selectively etching the A element (the group IIIA/IVA element) from MAX phases [13]. Gao et al. found that MXenes, like Ti_2_C, V_2_C and Ti_3_C_2_, are terminated by a mixture of O* and OH* terminal groups and exhibit metallic properties, indicating excellent charge transfer during HER catalysis [14]. The surface oxygen atoms of the MXenes function as the catalytic active sites for the HER with a reasonable interaction between hydrogen and MXenes. Seh and coworkers studied the catalytic performance of Mo_2_CT*_x_* and Ti_2_CT*_x_* MXenes and the results indicated that Mo_2_CT*_x_* possesses higher HER catalytic activity compared with Ti_2_CT*_x_* [15]. Apart from MXenes, many other TMCs have also been reported as potential HER catalysts. By theoretical calculations, Yu et al. systematically investigated the electrocatalytic performance of MC_2_ (M denotes transition metal of Ti, V, Nb, Ta, and Mo) [16]. The excellent thermodynamic stability and electronic conductivity of these structures can ensure rapid charge transfer during catalysis. In particular, NbC_2_, TaC_2_, and MoC_2_ exhibit efficient catalysis for HER according to the Volmer–Heyrovsky mechanism. These research findings indicate that the TMCs have great potential for HER catalysis.

With the development of synthesis strategies for catalysts, research in the field of single-atom catalysis has garnered extensive interest due to their remarkable activity [17,18,19,20]. Single-atom catalysts (SACs) exhibit significant charge transfer between the isolated metal atoms and coordination species of the supports, leading to high activity and selectivity [21]. Moreover, the active sites of SACs are atomically dispersed on the supports, which can enhance catalytic efficiency in a variety of reactions [22]. On account of high atomic utilization efficiency, catalytic activity and selectivity, Ru-based SACs show extremely attractive advantage for HER compared to Ru nanoparticles [23]. Liu et al. studied the HER catalysis ability of Ir SAC and the results indicated that Ir SAC possesses higher electrocatalytic activity than commercial Pt/C and Ir/C in HER. Its overpotential is notably lower than that of Pt/C and Ir/C catalysts by 26 and 3 mV, respectively [24]. Inspired by the great potential of TMCs to catalyze HER and the advantages of SACs, it is meaningful to investigate TMC-supported SACs.

In our recent work, a novel 2D tetragonal molybdenum carbide, Mo_3_C_2_, was theoretically found to be thermodynamically stable [25]. Although the 2D tetragonal Mo_3_C_2_ has the same stoichiometric ratio as the Mo_3_C_2_ MXene, its configuration is totally different from MXene. To our knowledge, well-known MXenes were first reported in 2011 by Naguib et al. [26], and the application of Mo_3_C_2_ MXene was first reported in 2017 by Li et al. in a study about CO_2_ capture and conversion [27]. Considering the good conductivity of the tetragonal Mo_3_C_2_, it is of great significance to investigate its catalytic performance in HER. In this work, using the density functional theory (DFT) method, we rationally imported transition-metal atoms in the C and Mo vacancies of the Mo_3_C_2_ sheet to design SACs to study their HER catalytic performance at different surface adsorption sites, where the transition-metal atoms refer to Ti, V, Cr, Mn, Fe, Co, Ni and Cu. Our theoretical study of the properties of the tetragonal Mo_3_C_2_ can offer researchers an incentive to explore synthesis methods for this material.

## 2. Computational Details

All the spin-polarized DFT calculations were carried out as implemented in the Vienna ab initio simulation package (VASP 5.4.4) [28,29]. The exchange correlation interactions were described by the Perdew–Burke–Ernzerhof (PBE) [30] functional within the generalized gradient approximation (GGA) [31]. The electron wave function was expanded into plane waves with an energy cutoff of 500 eV. The convergence criterion of 10^−5^ eV was used for the electron self-consistent loop and the force criterion of 0.02 eV/Å was adopted for the structure optimization. A vacuum distance larger than 15 Å (z direction) was employed to eliminate interactions between adjacent images. The long-range van der Waals interaction was described by the DFT-D3 method [32]. The studied SACs were constructed based on a 4 × 4 Mo_3_C_2_ supercell and a 4 × 4 × 1 *k*-point grid was chosen to sample the Brillouin zone. To examine the thermal stability of the catalysts, the ab initio molecular dynamics (AIMD) simulations were performed at 600 K with the Andersen thermostat [33] and *NVT* ensemble. The total duration of the AIMD simulations was 10 ps with a time step of 2 fs.

The defect formation energy in the tetragonal Mo_3_C_2_ supercell was calculated using the following equation:

(1) $$E_{\mathrm{form}}(V_{i})=E_{(4\times 4)-{\mathrm{Mo}}_{3}C_{2}-V_{i}}-E_{(4\times 4)-{\mathrm{Mo}}_{3}C_{2}}+n\mu_{i}$$

where $E_{(4\times 4)-{\mathrm{Mo}}_{3}C_{2}-V_{i}}$ and $E_{(4\times 4)-{\mathrm{Mo}}_{3}C_{2}}$ represent the energy of the Mo_3_C_2_ slab with and without vacancy defects, respectively. *μ*_i_ is the chemical potential of a type i atom, which is obtained by calculating the energy of an isolated type i atom. The value of *n* is the number of vacancies, which is 1 in this work.

The binding energy of transition metal atoms (TM = Ti, V, Cr, Mn, Fe, Co, Ni and Cu) located at the monovacancy of Mo_3_C_2_ was calculated via the following expression:

(2) $$E_{\mathrm{bind}}=E_{\mathrm{TM}+S}-E_{S}-E_{\mathrm{TM}}$$

where *E*_TM+S_ and *E*_S_ denote the energy of the defective Mo_3_C_2_ slab with and without embedded transition metal atoms, respectively. *E*_TM_ is the energy of the transition metal atom in its perfect bulk phase.

In acid electrolytes, the overall reaction scheme of HER can be described as:

H^+^(aq) + e^−^ + * → H* (3)

H* → 1/2H_2_(g) + * (4)

where the asterisk denotes the active sites on the surfaces of the catalysts, and aq and g stand for the aqueous and gas phases, respectively. The evaluation of HER catalytic activity is based on the Gibbs free energy change of hydrogen adsorption, which is defined as:

(5) $$\Delta G_{H*}=\Delta E_{H}+\Delta E_{\mathrm{ZPE}}-T\Delta S_{H}$$

where Δ*E*_H_ is the adsorption energy of hydrogen and can be calculated by the following equation:

(6) $$\Delta E_{H}=E_{\left(\mathrm{catalyst}+H\right)}-E_{\mathrm{catalyst}}-\frac{1}{2}E_{H_{2}}$$

in which *E*_catalyst+H_ and *E*_catalyst_ are the energies of the catalyst with and without adsorbed hydrogen, respectively. Δ*E*_ZPE_ is the change of zero-point energy and *E*_ZPE_ can be obtained by vibrational frequency calculation. It should be noted that the contributions from the catalysts to Δ*E*_ZPE_ can be ignored. Thus, Δ*E*_ZPE_ is calculated by the following equation:

(7) $$\Delta E_{\mathrm{ZPE}}=E_{\mathrm{ZPE}}^{H}-\frac{1}{2}E_{\mathrm{ZPE}}^{H_{2}}$$

where $E_{\mathrm{ZPE}}^{H}$ approximates to the zero-point energy of the adsorbed hydrogen atom and $E_{\mathrm{ZPE}}^{H_{2}}$ is the zero-point energy of gaseous H_2_. Moreover, *T* stands for the temperature (*T* = 298.15 K) and *T*Δ*S*_H_ is the change of vibrational entropy. Because the contributions from the catalysts to entropy are small and negligible, *T*Δ*S*_H_ can be regarded as the entropy difference between the adsorbed hydrogen atom and gaseous hydrogen. The entropy of H_2_ in the gas phase at 298.15 K is approximately 0.40 eV [34].

## 3. Results and Discussion

### 3.1. Structure, Stability and Active Sites

**Figure 1:**
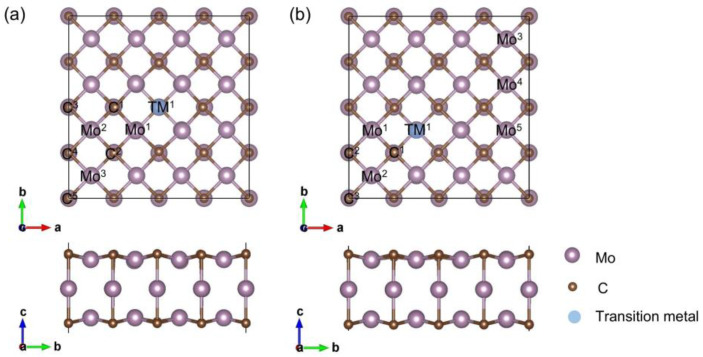
The structure and active sites of Mo_3_C_2_-based single-atom catalysts, where transition-metal atoms (Ti, V, Cr, Mn, Fe, Co, Ni and Cu) are located at a (**a**) C-vacancy or (**b**) Mo_surf_-vacancy.

First of all, the formation of vacancy and binding of transition-metal atoms were studied. Considering that vacancies are more likely to form on the surface of the material, the vacancy formation energies (*E*_form_) of C and Mo_surf_ (surface Mo) monovacancies were calculated, and the corresponding formation energies are 7.67 and 10.99 eV, respectively. The binding energies (*E*_bind_) of transition-metal atoms located at C or Mo_surf_ monovacancy were calculated, and the results are listed in Table 1. The binding energies of all the transition-metal atoms at the C vacancy range from 0.78 to 2.81 eV, while those at Mo_surf_ vacancy range from −5.19 to −1.27. The Cr atom at the C vacancy exhibits the biggest binding energy (2.81 eV), whereas the Ti atom at the Mo_surf_ vacancy exhibits the smallest binding energy (−5.19 eV). Although the Mo_surf_ vacancy has a higher formation energy compared with the C vacancy, it exhibits negative binding energies for all of the studied transition-metal atoms. Thus, in this work, both C and Mo_surf_ vacancies are considered to be substituted by transition-metal atoms. The lattice constants of (4 × 4)-Mo_3_C_2_ with C and Mo_surf_ vacancies are 11.777 and 11.767 Å, respectively, slightly lower than that of (4 × 4)-Mo_3_C_2_ (11.778 Å). Figure 1 displays the structural configurations of Mo_3_C_2_-based SACs associated with considered active sites for HER, and Figures S1 and S2 (Supplementary Materials) present the optimized structures of the SACs. After optimizing the constructed models of SACs, it is found that the transition-metal atoms are significantly higher than the surface of the pristine Mo_3_C_2_ when located at the C vacancy (Figure S1). This can be attributed to the fact that the radii of transition-metal atoms are larger than that of carbon atom. For SACs with transition-metal atoms stabilized at the Mo vacancy, the imported transition-metal atoms are almost at the same height as the surface of the pristine Mo_3_C_2_ (Figure S2). The SACs are labeled as TM@(4 × 4)-Mo_3_C_2_-V_C_ and TM@(4 × 4)-Mo_3_C_2_-V_surf-Mo_, as shown in Figures S1 and S2, respectively. For TM@(4 × 4)-Mo_3_C_2_-V_C_ and TM@(4 × 4)-Mo_3_C_2_-V_surf-Mo_, the imported transition-metal atoms are surrounded by five Mo atoms and four C atoms, respectively. In addition, the calculated lattice constants of TM@(4 × 4)-Mo_3_C_2_-V_C_ and TM@(4 × 4)-Mo_3_C_2_-V_surf-Mo_ are listed in Tables S1 and S2, respectively. The lattice constants of all the TM@(4 × 4)-Mo_3_C_2_-V_C_ are bigger than that of (4 × 4)-Mo_3_C_2_, while the lattice constants of most TM@(4 × 4)-Mo_3_C_2_-V_C_ are smaller than that of (4 × 4)-Mo_3_C_2_. Furthermore, the thermal stability of the discussed structures was examined by AIMD simulations at 600 K with a time step of 2 fs, and the results are shown in Figures S3 and S4. During the AIMD simulations, the total energies of all the studied systems fluctuated around the equilibrium positions. After performing simulations for 10 ps, all the configurations displayed no obvious reconstruction. These results suggest that all the SACs modelled in this work are thermally stable at high temperature.

The active sites for HER are marked in Figure 1. For the SACs with transition-metal atoms (Ti, V, Cr, Mn, Fe, Co, Ni and Cu) located at the C-vacancy, the tops of transition-metal atoms (site TM^1^), Mo atoms (sites Mo^1^, Mo^2^ and Mo^3^) and C atoms (sites C^1^, C^2^, C^3^, C^4^ and C^5^) are selected as the possible active sites for HER. When the transition-metal atoms are located at the Mo_surf_-vacancy, one site above the transition-metal atoms (site TM^1^), three sites above the C atoms (sites C^1^, C^2^ and C^3^) and five sites above the Mo atoms (sites Mo^1^, Mo^2^, Mo^3^, Mo^4^ and Mo^5^) are selected as possible active sites.

### 3.2. Electronic Properties

**Figure 2:**
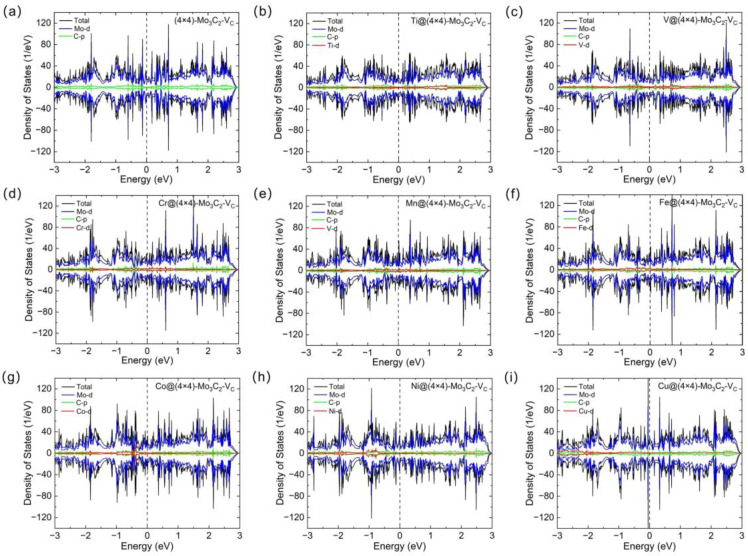
Total and partial density of states of (**a**) (4 × 4)-Mo_3_C_2_-V_C_, (**b**) Ti@(4 × 4)-Mo_3_C_2_-V_C_, (**c**) V@(4 × 4)-Mo_3_C_2_-V_C_, (**d**) Cr@(4 × 4)-Mo_3_C_2_-V_C_, (**e**) Mn@(4 × 4)-Mo_3_C_2_-V_C_, (**f**) Fe@(4 × 4)-Mo_3_C_2_-V_C_, (**g**) Co@(4 × 4)-Mo_3_C_2_-V_C_, (**h**) Ni@(4 × 4)-Mo_3_C_2_-V_C_ and (**i**) Cu@(4 × 4)-Mo_3_C_2_-V_C_. The Fermi level is set to 0 eV and marked with the dashed line.

**Figure 3:**
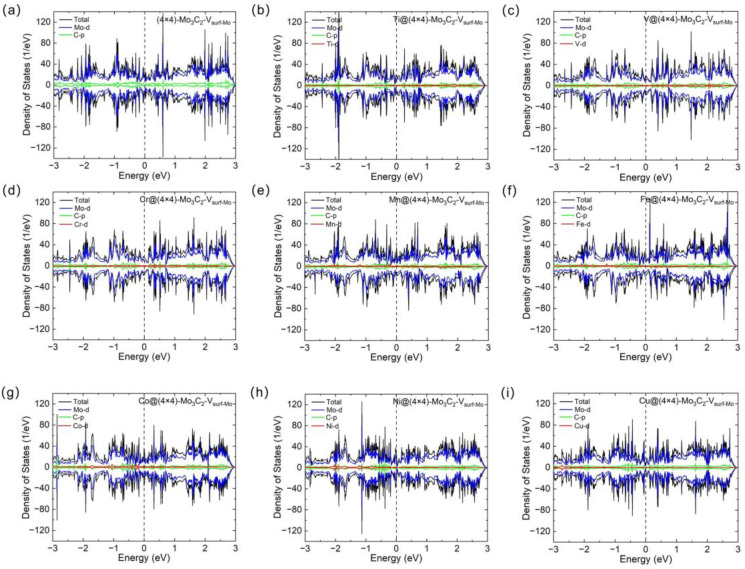
Total and partial density of states of (**a**) (4 × 4)-Mo_3_C_2_-V_surf-Mo_, (**b**) Ti@(4 × 4)-Mo_3_C_2_-V_surf-Mo_, (**c**) V@(4 × 4)-Mo_3_C_2_-V_surf-Mo_, (**d**) Cr@(4 × 4)-Mo_3_C_2_-V_surf-Mo_, (**e**) Mn@(4 × 4)-Mo_3_C_2_-V_surf-Mo_, (**f**) Fe@(4 × 4)-Mo_3_C_2_-V_surf-Mo_, (**g**) Co@(4 × 4)-Mo_3_C_2_-V_surf-Mo_, (**h**) Ni@(4 × 4)-Mo_3_C_2_-V_surf-Mo_ and (**i**) Cu@(4 × 4)-Mo_3_C_2_-V_surf-Mo_. The Fermi level is set to 0 eV and marked with the dashed line.

The catalytic properties are closely related to the electronic structures of catalysts [35,36]. Thus, the density of states (DOS) of (4 × 4)-Mo_3_C_2_ with C/Mo_surf_ vacancy, TM@(4 × 4)-Mo_3_C_2_-V_C_ and TM@(4 × 4)-Mo_3_C_2_-V_surf-Mo_ were calculated. As shown in Figure 2a and Figure 3a, the Mo_3_C_2_ slab with C/Mo_surf_ monovacancy exhibits metallic conductivity with obvious DOS near the Fermi level. Meanwhile, all the TM@(4 × 4)-Mo_3_C_2_-V_C_ and TM@(4 × 4)-Mo_3_C_2_-V_surf-Mo_ are also metallic (Figure 2b–i and Figure 3b–i). According to the projected density of states (PDOS), the DOS near the Fermi level of the studied materials are mainly contributed by the Mo-d orbitals, while a small contribution is from C-p and TM-d orbitals. For the DOS of all the structures, there are obvious overlaps near the Fermi energy between the Mo-d and C-p orbitals, indicating p-d orbital hybridizations between C and Mo atoms. In general, the good electronic conduction of the Mo_3_C_2_-based SACs can ensure excellent charge transfer in HER.

### 3.3. Catalytic Activity for Hydrogen Evolution Reaction

Typically, the whole HER process can be depicted by a three-state diagram, which includes the initial state H^+^ + e^−^, the intermediate state H* and the final product H_2_. In this work, we analyze the HER catalytic ability of TM@(4 × 4)-Mo_3_C_2_-V_C_ and TM@(4 × 4)-Mo_3_C_2_-V_surf-Mo_ at different active sites by calculating the Gibbs free energy difference (Δ*G*_H*_) between the intermediate state H* and the final product H_2_, which is regarded as the most important descriptor to assess the HER catalytic activity of an electrocatalyst. Obviously, a smaller absolute value of Δ*G*_H*_ implies a smaller energy barrier of HER. In general, catalysts with |Δ*G*_H*_| smaller than 0.2 eV are considered to be excellent HER catalysts and the ideal value of Δ*G*_H*_ is 0 eV [37].

The calculation details for Δ*G*_H*_ are shown in Tables S3–S18, including energies (*E*), zero-point energies (*E*zpe), vibrational entropy (*TS*_H_), Gibbs free energies (*G*) and Δ*G*_H*_ of every SAC at different adsorption sites. It can be found that *E*zpe values are closely related to the atomic species that adsorb hydrogen atoms. The *E*zpe values for SACs with hydrogen atoms adsorbed on the top of C atoms (around 0.25 eV) are larger than the counterparts adsorbing H on the top of Mo or imported transition-metal atoms (less than 0.20 eV). In addition, the *TS*_H_ values for SACs with hydrogen atoms adsorbed on the top of C and Mo atoms are 0.01 eV, far less than half that of gaseous hydrogen (0.20 eV). Overall, *E*zpe has a greater impact on free energy than *TS*_H_.

The Δ*G*_H*_ values of TM@(4 × 4)-Mo_3_C_2_-V_C_ at different sites are listed in Table 2. For each TM@(4 × 4)-Mo_3_C_2_-V_C_, the Δ*G*_H*_ at the adsorption sites on the top of C atoms are smaller than those on the top of Mo atoms except for the Mo^1^ site. This is related to the lower adsorption heights of hydrogen atoms at the top of C atoms compared with Mo atoms, which indicates a higher adsorption strength. After optimizing the structures of TM@(4 × 4)-Mo_3_C_2_-V_C_ with a hydrogen atom adsorbed on the Mo^1^ site, the hydrogen atom is not located directly above the Mo atom, but moves a small distance towards the imported transition-metal atom, suggesting that the imported transition-metal atoms have a considerable impact on the adsorption of hydrogen atoms. Due to the fact that the Mo^1^ site is closer to the imported transition-metal atom compared with the Mo^2^ and Mo^3^ sites, the Δ*G*_H*_ at the Mo^1^ site differs significantly from the Mo^2^ and Mo^3^ sites. Overall, the Δ*G*_H*_ at the adsorption sites on the top of C atoms are closer to zero than at other sites. For all the TM@(4 × 4)-Mo_3_C_2_-V_C_, the Mo^2^ site exhibits the largest value of Δ*G*_H*_. Apart from (Cr, Mn and Fe)@(4 × 4)-Mo_3_C_2_-V_C_, other TM@(4 × 4)-Mo_3_C_2_-V_C_ exhibit high HER catalytic activity. Specifically, (Co and Cu)@(4 × 4)-Mo_3_C_2_-V_C_ show great HER catalytic activity at sites C^1^, C^2^, C^3^, C^4^, C^5^, Mo^1^ and TM^1^. Compared with (Co and Cu)@(4 × 4)-Mo_3_C_2_-V_C_, (Ti, V and Ni)@(4 × 4)-Mo_3_C_2_-V_C_ have fewer active sites with |Δ*G*_H*_| smaller than 0.2 eV. It should be noted that the Δ*G*_H*_ of Ti@(4 × 4)-Mo_3_C_2_-V_C_ at site C^5^ is zero, suggesting the best HER catalytic capacity.

Table 3 lists the Δ*G*_H*_ values of different adsorption sites for TM@(4 × 4)-Mo_3_C_2_-V_surf-Mo_. Similar to TM@(4 × 4)-Mo_3_C_2_-V_C_, adsorption sites on the top of C atoms display smaller |Δ*G*_H*_| values compared with other sites. The free energies for all the sites on the top of Mo atoms and imported transition-metal atoms are larger than 0.30 eV, indicating inefficient catalytic performance. The adsorption site TM^1^ exhibits the largest value of Δ*G*_H*_, which is different from SACs related to the C vacancy. For all the TM@(4 × 4)-Mo_3_C_2_-V_surf-Mo_, every site on the top of C atoms meets the condition of |Δ*G*_H*_| < 0.2 eV. Particularly, the Δ*G*_H*_ of Ti@(4 × 4)-Mo_3_C_2_-V_surf-Mo_ at the C^2^ site, Fe@(4 × 4)-Mo_3_C_2_-V_surf-Mo_ at C^2^ and C^3^ sites, and Ni@(4 × 4)-Mo_3_C_2_-V_surf-Mo_ at the C^3^ site are calculated as zero. On the whole, we can conclude that every TM@(4 × 4)-Mo_3_C_2_-V_surf-Mo_ can act as an excellent HER catalyst.

## 4. Summary

In summary, we have designed a series of SACs by importing transition-metal atoms in the C and Mo vacancies of 2D tetragonal Mo_3_C_2_ and examined the possibility of using these SACs as HER catalysts with the first-principles computations. The results suggest that all the SACs possess good thermal stability and metallic conductivity. The Gibbs free energy change of hydrogen adsorption at different active sites predicts that, apart from SACs with Cr, Mn and Fe located at C vacancies, all of the other SACs possess catalytic ability for HER. This work indicates that the tetragonal Mo_3_C_2_-based SACs are expected to be efficient and economical candidates for HER catalysts applied in water splitting.

## Figures and Tables

**Table 1:** The binding energies *E*_bind_ (eV) of transition-metal atoms substituted C or Mo_surf_ monovacancies of (4 × 4)-Mo_3_C_2_.

	Ti	V	Cr	Mn	Fe	Co	Ni	Cu
C monovacancy	1.09	2.14	2.81	2.36	2.01	1.43	0.83	0.78
Mo_surf_ monovacancy	−5.19	−4.39	−3.27	−3.25	−2.79	−2.43	−2.21	−1.27

**Table 2:** Calculated Gibbs free energies of the hydrogen adsorptions at different active sites for TM@(4 × 4)-Mo_3_C_2_-V_C_. The units are in electron volts.

	C^1^	C^2^	C^3^	C^4^	C^5^	Mo^1^	Mo^2^	Mo^3^	TM^1^
Ti@(4 × 4)-Mo_3_C_2_-V_C_	−0.24	−0.06	0.08	−0.14	0.00	−0.42	0.34	0.24	0.28
V@(4 × 4)-Mo_3_C_2_-V_C_	−0.40	−0.11	0.05	−0.31	−0.03	−0.36	0.34	0.23	0.15
Cr@(4 × 4)-Mo_3_C_2_-V_C_	−0.65	−0.62	−0.70	−0.73	−0.75	−1.07	−0.46	−0.46	−0.69
Mn@(4 × 4)-Mo_3_C_2_-V_C_	−1.01	−0.98	−1.06	−1.09	−1.08	−1.38	−0.78	−0.78	−1.03
Fe@(4 × 4)-Mo_3_C_2_-V_C_	−0.55	−0.51	−0.56	−0.60	−0.61	−0.83	−0.28	−0.28	−0.55
Co@(4 × 4)-Mo_3_C_2_-V_C_	0.18	0.15	0.08	0.06	0.05	−0.14	0.38	0.34	0.04
Ni@(4 × 4)-Mo_3_C_2_-V_C_	0.21	0.21	0.10	0.08	0.05	−0.12	0.36	0.34	0.08
Cu@(4 × 4)-Mo_3_C_2_-V_C_	0.18	0.19	0.08	0.05	0.06	−0.13	0.33	0.33	0.16

**Table 3:** Calculated Gibbs free energies of the hydrogen adsorptions at different active sites for TM@(4 × 4)-Mo_3_C_2_-V_surf-Mo_. The units are in electron volts.

	C^1^	C^2^	C^3^	Mo^1^	Mo^2^	Mo^3^	Mo^4^	Mo^5^	TM^1^
Ti@(4 × 4)-Mo_3_C_2_-V_surf-Mo_	0.09	0.00	−0.04	0.37	0.39	0.39	0.40	0.40	0.80
V@(4 × 4)-Mo_3_C_2_-V_surf-Mo_	0.01	−0.03	−0.02	0.37	0.36	0.35	0.38	0.37	0.67
Cr@(4 × 4)-Mo_3_C_2_-V_surf-Mo_	−0.04	−0.02	−0.01	0.37	0.37	0.36	0.38	0.37	0.50
Mn@(4 × 4)-Mo_3_C_2_-V_surf-Mo_	−0.06	−0.01	−0.02	0.38	0.35	0.33	0.39	0.38	0.61
Fe@(4 × 4)-Mo_3_C_2_-V_surf-Mo_	−0.10	0.00	0.00	0.39	0.36	0.33	0.40	0.39	0.63
Co@(4 × 4)-Mo_3_C_2_-V_surf-Mo_	−0.13	−0.01	−0.02	0.40	0.35	0.31	0.39	0.38	0.69
Ni@(4 × 4)-Mo_3_C_2_-V_surf-Mo_	−0.07	0.02	0.00	0.40	0.37	0.31	0.42	0.38	0.99
Cu@(4 × 4)-Mo_3_C_2_-V_surf-Mo_	−0.07	0.01	−0.06	0.36	0.37	0.33	0.41	0.39	1.25

## Data Availability

The original contributions presented in the study are included in the article/Supplementary Materials; further inquiries can be directed to the corresponding authors.

## Supplementary Materials

The following supporting information can be downloaded at: https://www.mdpi.com/article/10.3390/ma17246134/s1, Figure S1. Top and side views of the optimized configurations of (a) Ti@(4 × 4)-Mo_3_C_2_-C_v_, (b) V@(4 × 4)-Mo_3_C_2_-C_v_, (c) Cr@(4 × 4)-Mo_3_C_2_-C_v_, (d) Mn@(4 × 4)-Mo_3_C_2_-C_v_, (e) Fe@(4 × 4)-Mo_3_C_2_-C_v_, (f) Co@(4 × 4)-Mo_3_C_2_-C_v_, (g) Ni@(4 × 4)-Mo_3_C_2_-C_v_ and (h) Cu@(4 × 4)-Mo_3_C_2_-C_v_. Figure S2. Top and side views of the optimized configurations of (a) Ti@(4 × 4)-Mo_3_C_2_-C_surf-Mo_, (b) V@(4 × 4)-Mo_3_C_2_-C_surf-Mo_, (c) Cr@(4 × 4)-Mo_3_C_2_-C_surf-Mo_, (d) Mn@(4 × 4)-Mo_3_C_2_-C_surf-Mo_, (e) Fe@(4 × 4)-Mo_3_C_2_-C_surf-Mo_, (f) Co@(4 × 4)-Mo_3_C_2_-C_surf-Mo_, (g) Ni@(4 × 4)-Mo_3_C_2_-C_surf-Mo_ and (h) Cu@(4 × 4)-Mo_3_C_2_-C_surf-Mo_. Figure S3. Energies as a function of time of (a) Ti@(4 × 4)-Mo_3_C_2_-C_v_, (b) V@(4 × 4)-Mo_3_C_2_-C_v_, (c) Cr@(4 × 4)-Mo_3_C_2_-C_v_, (d) Mn@(4 × 4)-Mo_3_C_2_-C_v_, (e) Fe@(4 × 4)-Mo_3_C_2_-C_v_, (f) Co@(4 × 4)-Mo_3_C_2_-C_v_, (g) Ni@(4 × 4)-Mo_3_C_2_-C_v_ and (h) Cu@(4 × 4)-Mo_3_C_2_-C_v_ during the AIMD simulations (inset: the configurations after 10 ps AIMD simulations). Figure S4. Energies as a function of time of (a) Ti@(4 × 4)-Mo_3_C_2_-C_surf-Mo_, (b) V@(4 × 4)-Mo_3_C_2_-C_surf-Mo_, (c) Cr@(4 × 4)-Mo_3_C_2_-C_surf-Mo_, (d) Mn@(4 × 4)-Mo_3_C_2_-C_surf-Mo_, (e) Fe@(4 × 4)-Mo_3_C_2_-C_surf-Mo_, (f) Co@(4 × 4)-Mo_3_C_2_-C_surf-Mo_, (g) Ni@(4 × 4)-Mo_3_C_2_-C_surf-Mo_ and (h) Cu@(4 × 4)-Mo_3_C_2_-C_surf-Mo_ during the AIMD simulations (inset: the configurations after 10 ps AIMD simulations). Table S1. The lattice constants (Å) of TM@(4 × 4)-Mo_3_C_2_-V_C_. Table S2. The lattice constants (Å) of TM@(4 × 4)-Mo_3_C_2_-V_surf-Mo_. Table S3. Calculated energies (*E*), zero-point energies (*E*zpe), vibrational entropy (*TS*_H_), Gibbs free energies (*G*) and Gibbs free energy differences (Δ*G*) of Ti@(4 × 4)-Mo_3_C_2_-V_C_ at different adsorption sites. Table S4. Calculated energies (*E*), zero-point energies (*E*zpe), vibrational entropy (*TS*_H_), Gibbs free energies (*G*) and Gibbs free energy differences (Δ*G*) of V@(4 × 4)-Mo_3_C_2_-V_C_ at different adsorption sites. Table S5. Calculated energies (*E*), zero-point energies (*E*zpe), vibrational entropy (*TS*_H_), Gibbs free energies (*G*) and Gibbs free energy differences (Δ*G*) of Cr@(4 × 4)-Mo_3_C_2_-V_C_ at different adsorption sites. Table S6. Calculated energies (*E*), zero-point energies (*E*zpe), vibrational entropy (*TS*_H_), Gibbs free energies (*G*) and Gibbs free energy differences (Δ*G*) of Mn@(4 × 4)-Mo_3_C_2_-V_C_ at different adsorption sites. Table S7. Calculated energies (*E*), zero-point energies (*E*zpe), vibrational entropy (*TS*_H_), Gibbs free energies (*G*) and Gibbs free energy differences (Δ*G*) of Fe@(4 × 4)-Mo_3_C_2_-V_C_ at different adsorption sites. Table S8. Calculated energies (*E*), zero-point energies (*E*zpe), vibrational entropy (*TS*_H_), Gibbs free energies (*G*) and Gibbs free energy differences (Δ*G*) of Co@(4 × 4)-Mo_3_C_2_-V_C_ at different adsorption sites. Table S9. Calculated energies (*E*), zero-point energies (*E*zpe), vibrational entropy (*TS*_H_), Gibbs free energies (*G*) and Gibbs free energy differences (Δ*G*) of Ni@(4 × 4)-Mo_3_C_2_-V_C_ at different adsorption sites. Table S10. Calculated energies (*E*), zero-point energies (*E*zpe), vibrational entropy (*TS*_H_), Gibbs free energies (*G*) and Gibbs free energy differences (Δ*G*) of Cu@(4 × 4)-Mo_3_C_2_-V_C_ at different adsorption sites. Table S11. Calculated energies (*E*), zero-point energies (*E*zpe), vibrational entropy (*TS*_H_), Gibbs free energies (*G*) and Gibbs free energy differences (Δ*G*) of Ti@(4 × 4)-Mo_3_C_2_-V_surf-Mo_ at different adsorption sites. Table S12. Calculated energies (*E*), zero-point energies (*E*zpe), vibrational entropy (*TS*_H_), Gibbs free energies (*G*) and Gibbs free energy differences (Δ*G*) of V@(4 × 4)-Mo_3_C_2_-V_surf-Mo_ at different adsorption sites. Table S13. Calculated energies (*E*), zero-point energies (*E*zpe), vibrational entropy (*TS*_H_), Gibbs free energies (*G*) and Gibbs free energy differences (Δ*G*) of Cr@(4 × 4)-Mo_3_C_2_-V_surf-Mo_ at different adsorption sites. Table S14. Calculated energies (*E*), zero-point energies (*E*zpe), vibrational entropy (*TS*_H_), Gibbs free energies (*G*) and Gibbs free energy differences (Δ*G*) of Mn@(4 × 4)-Mo_3_C_2_-V_surf-Mo_ at different adsorption sites. Table S15. Calculated energies (*E*), zero-point energies (*E*zpe), vibrational entropy (*TS*_H_), Gibbs free energies (*G*) and Gibbs free energy differences (Δ*G*) of Fe@(4 × 4)-Mo_3_C_2_-V_surf-Mo_ at different adsorption sites. Table S16. Calculated energies (*E*), zero-point energies (*E*zpe), vibrational entropy (*TS*_H_), Gibbs free energies (*G*) and Gibbs free energy differences (Δ*G*) of Co@(4 × 4)-Mo_3_C_2_-V_surf-Mo_ at different adsorption sites. Table S17. Calculated energies (*E*), zero-point energies (*E*zpe), vibrational entropy (*TS*_H_), Gibbs free energies (*G*) and Gibbs free energy differences (Δ*G*) of Ni@(4 × 4)-Mo_3_C_2_-V_surf-Mo_ at different adsorption sites. Table S18. Calculated energies (*E*), zero-point energies (*E*zpe), vibrational entropy (*TS*_H_), Gibbs free energies (*G*) and Gibbs free energy differences (Δ*G*) of Cu@(4 × 4)-Mo_3_C_2_-V_surf-Mo_ at different adsorption sites.
