# Supplementary material for: Theoretical Investigation of Single-Atom Catalysts for Hydrogen Evolution Reaction Based on Two-Dimensional Tetragonal Mo3C2
Source: Materials (Basel). 2024 Dec 15;17(24):6134. doi: 10.3390/ma17246134 (PMC11678005; doi:10.3390/ma17246134)
Supplement: Supplementary file 1 [file materials-17-06134-s001.zip › materials-3342046-supplementary.pdf]

Electronic Supplementary Information

Theoretical Investigation of Single-Atom Catalysts for Hydrogen Evolution Reaction Based on Two-Dimensional Tetragonal Mo_3C_2

Bo Xue [1,*], Qingfeng Zeng [2,3,4], Shuyin Yu [2,3] and Kehe Su [5,*]

[1] School of Physical Science and Technology, Northwestern Polytechnical University, Xi'an 710129, China
[2] MSEA International Institute for Materials Genome, Langfang 065500, China; zengqf@dianyunkeji.com (Q.Z.); yusy@dianyunkeji.com (S.Y.)
[3] Particle Cloud Biotechnology (Hangzhou) Co., Ltd., Hangzhou 310018, China
[4] Science and Technology on Thermostructural Composite Materials Laboratory, Northwestern Polytechnical University, Xi'an 710072, China
[5] School of Chemistry and Chemical Engineering, Northwestern Polytechnical University, Xi'an 710129, China
* Correspondence: xuebo@mail.nwpu.edu.cn (B.X.); sukehe@nwpu.edu.cn (K.S.)

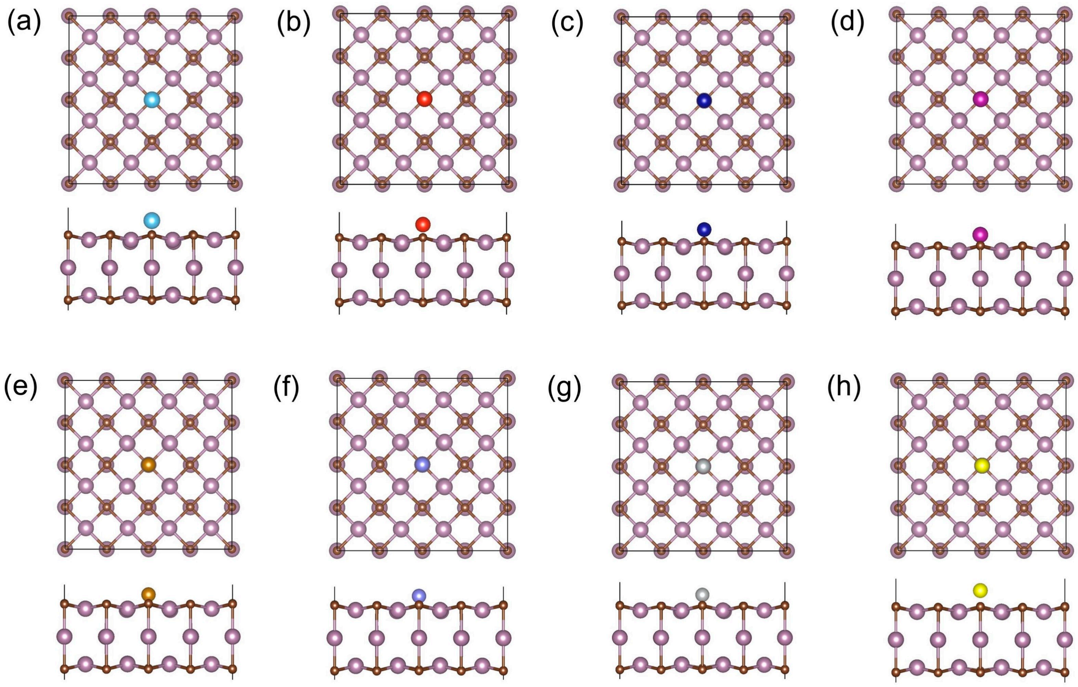

Figure S1. Top and side views of the optimized configurations of (**a**) Ti@(4 × 4)-Mo_3C_2-C_v, (**b**) V@(4 × 4)-Mo_3C_2-C_v, (**c**) Cr@(4 × 4)-Mo_3C_2-C_v, (**d**) Mn@(4 × 4)-Mo_3C_2-C_v, (**e**) Fe@(4 × 4)-Mo_3C_2-C_v, (**f**) Co@(4 × 4)-Mo_3C_2-C_v, (**g**) Ni@(4 × 4)-Mo_3C_2-C_v and (**h**) Cu@(4 × 4)-Mo_3C_2-C_v.

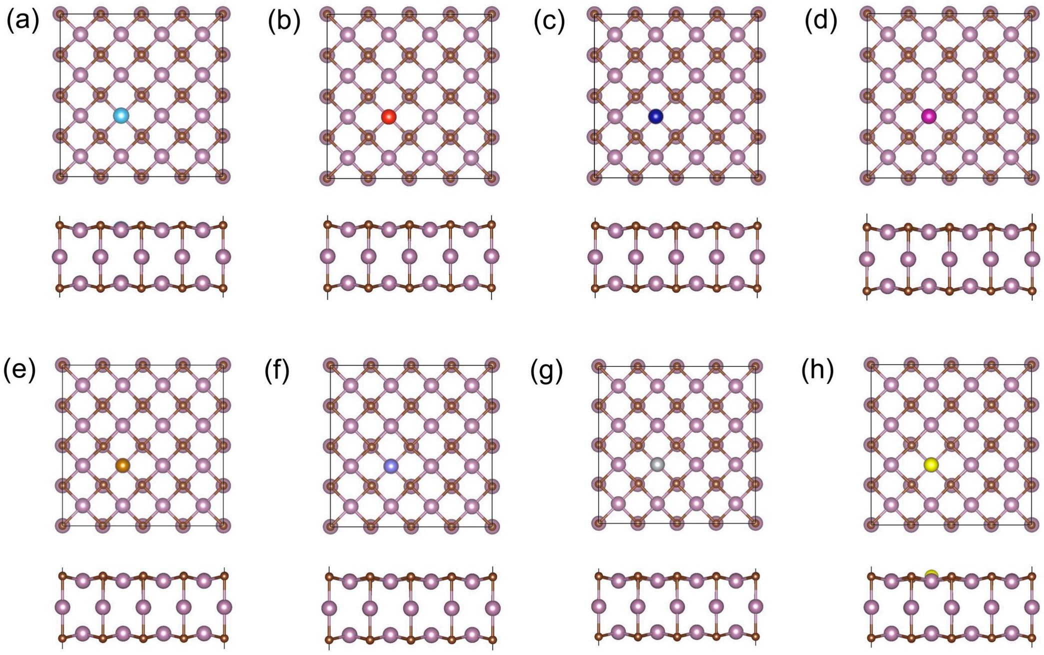

Figure S2. Top and side views of the optimized configurations of (**a**) Ti@(4 × 4)-Mo_3C_2-$C_{surf\text{-}Mo}$, (**b**) V@(4 × 4)-Mo_3C_2-$C_{surf\text{-}Mo}$, (**c**) Cr@(4 × 4)-Mo_3C_2-$C_{surf\text{-}Mo}$, (**d**) Mn@(4 × 4)-Mo_3C_2-$C_{surf\text{-}Mo}$, (**e**) Fe@(4 × 4)-Mo_3C_2-$C_{surf\text{-}Mo}$, (**f**) Co@(4 × 4)-Mo_3C_2-$C_{surf\text{-}Mo}$, (**g**) Ni@(4 × 4)-Mo_3C_2-$C_{surf\text{-}Mo}$ and (**h**) Cu@(4 × 4)-Mo_3C_2-$C_{surf\text{-}Mo}$.

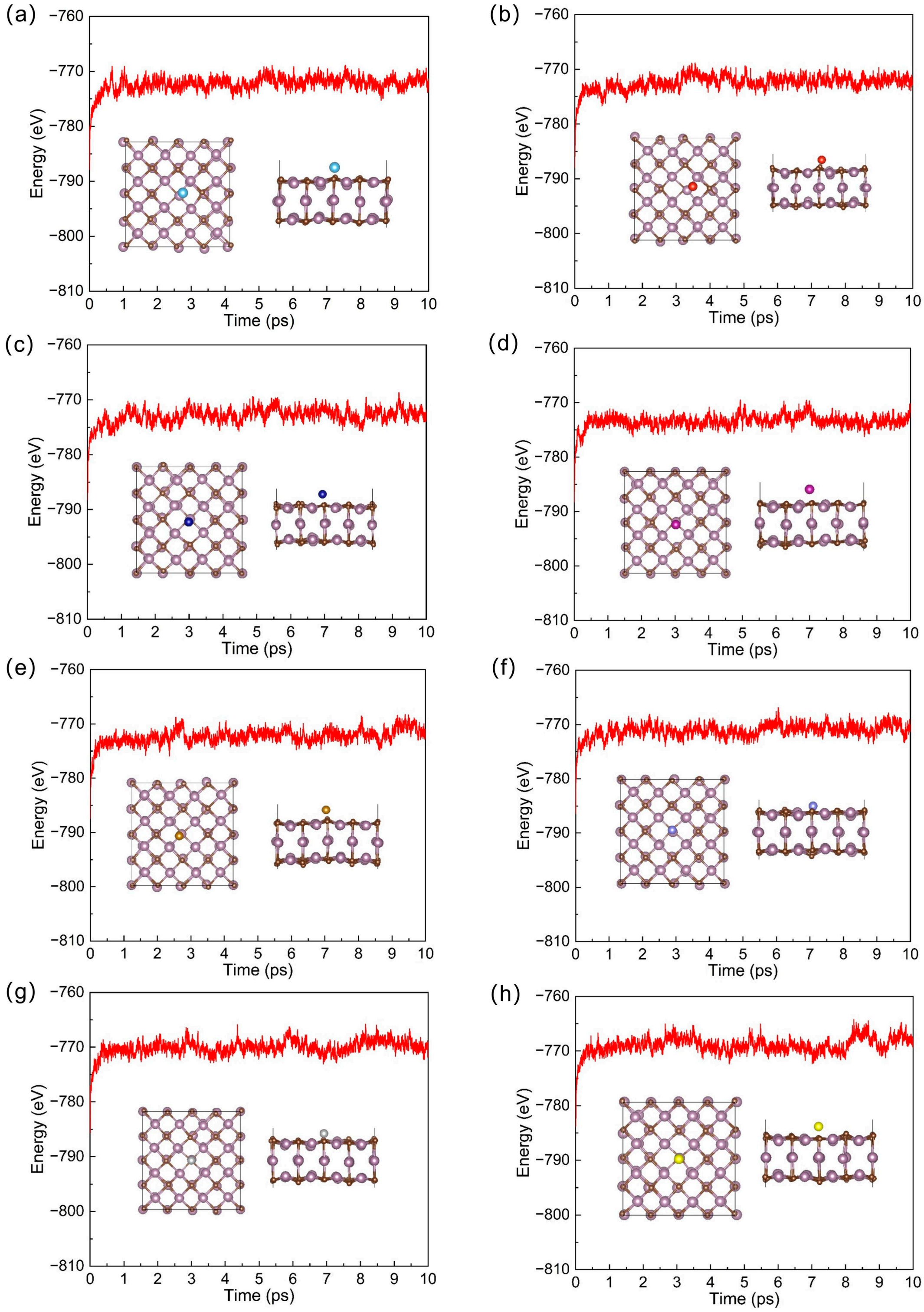

Figure S3. Energies as a function of time of (**a**) Ti@(4 × 4)-Mo_3C_2-C_v, (**b**) V@(4 × 4)-Mo_3C_2-C_v, (**c**) Cr@(4 × 4)-Mo_3C_2-C_v, (**d**) Mn@(4 × 4)-Mo_3C_2-C_v, (**e**) Fe@(4 × 4)-Mo_3C_2-C_v, (**f**) Co@(4 × 4)-Mo_3C_2-C_v, (**g**) Ni@(4 × 4)-Mo_3C_2-C_v and (**h**) Cu@(4 × 4)-Mo_3C_2-C_v during the AIMD simulations (inset: the configurations after 10 ps AIMD simulations).

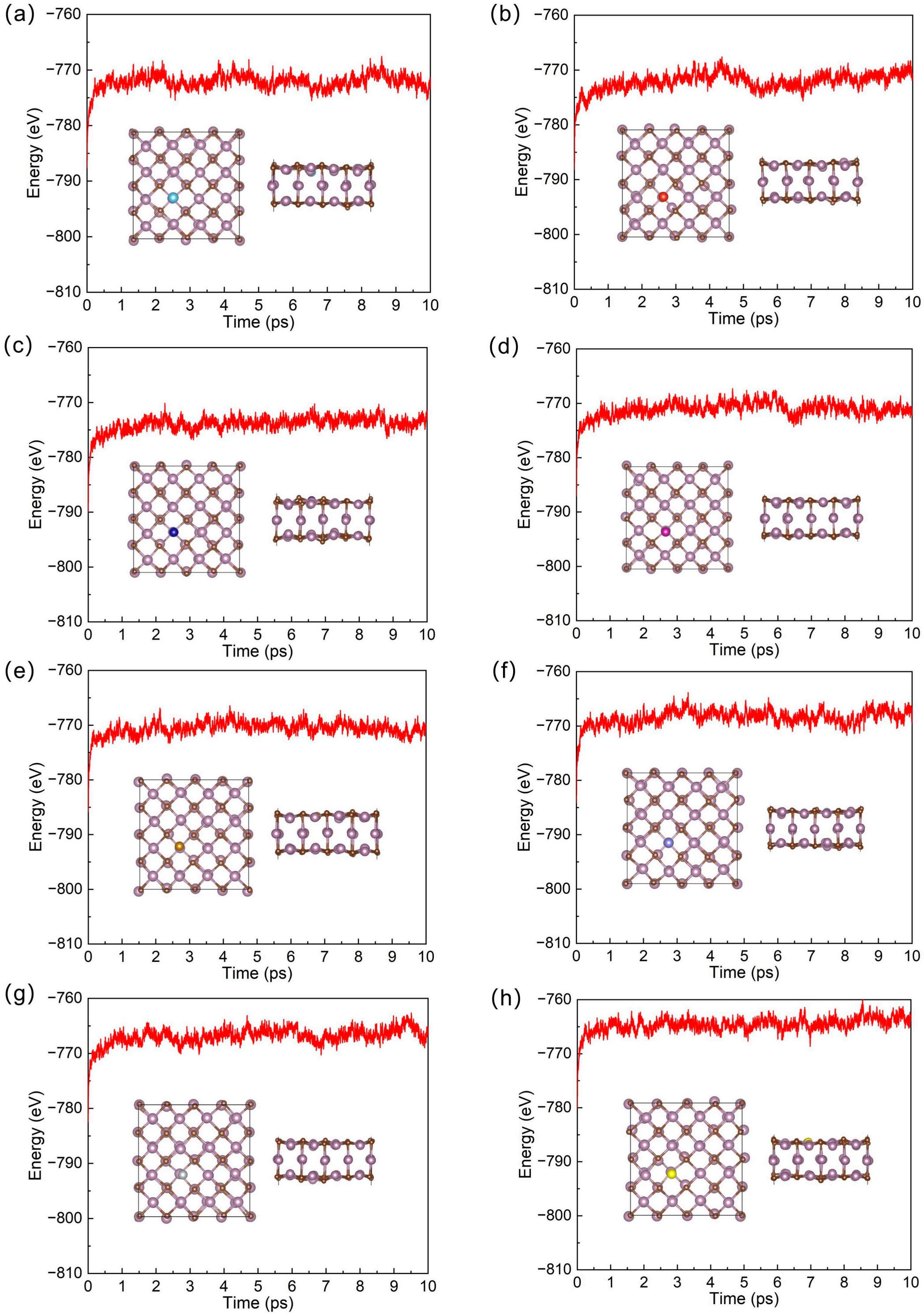

Figure S4. Energies as a function of time of (**a**) Ti@(4 × 4)-Mo_3C_2-$C_{surf\text{-}Mo}$, (**b**) V@(4 × 4)-Mo_3C_2-$C_{surf\text{-}Mo}$, (**c**) Cr@(4 × 4)-Mo_3C_2-$C_{surf\text{-}Mo}$, (**d**) Mn@(4 × 4)-Mo_3C_2-$C_{surf\text{-}Mo}$, (**e**) Fe@(4 × 4)-Mo_3C_2-$C_{surf\text{-}Mo}$, (**f**) Co@(4 × 4)-Mo_3C_2-$C_{surf\text{-}Mo}$, (**g**) Ni@(4 × 4)-Mo_3C_2-$C_{surf\text{-}Mo}$ and (**h**) Cu@(4 × 4)-Mo_3C_2-$C_{surf\text{-}Mo}$ during the AIMD simulations (inset: the configurations after 10 ps AIMD simulations).

Table S1. The lattice constants (Å) of TM@(4 × 4)-Mo_3C_2-V_C.

Structure	$a = b$/Å
Ti@(4 × 4)-Mo_3C_2-V_C	11.785
V@(4 × 4)-Mo_3C_2-V_C	11.785
Cr@(4 × 4)-Mo_3C_2-V_C	11.786
Mn@(4 × 4)-Mo_3C_2-V_C	11.789
Fe@(4 × 4)-Mo_3C_2-V_C	11.790
Co@(4 × 4)-Mo_3C_2-V_C	11.794
Ni@(4 × 4)-Mo_3C_2-V_C	11.793
Cu@(4 × 4)-Mo_3C_2-V_C	11.784

Table S2. The lattice constants (Å) of TM@(4 × 4)-Mo_3C_2-$V_{surf\text{-}Mo}$.

Structure	$a = b$/Å
Ti@(4 × 4)-Mo_3C_2-$V_{surf\text{-}Mo}$	11.779
V@(4 × 4)-Mo_3C_2-$V_{surf\text{-}Mo}$	11.764
Cr@(4 × 4)-Mo_3C_2-$V_{surf\text{-}Mo}$	11.757
Mn@(4 × 4)-Mo_3C_2-$V_{surf\text{-}Mo}$	11.760
Fe@(4×4)-Mo_3C_2-$V_{surf\text{-}Mo}$	11.757
Co@(4 × 4)-Mo_3C_2-$V_{surf\text{-}Mo}$	11.755
Ni@(4 × 4)-Mo_3C_2-$V_{surf\text{-}Mo}$	11.759
Cu@(4 × 4)-Mo_3C_2-$V_{surf\text{-}Mo}$	11.771

Table S3. Calculated energies (*E*), zero-point energies (*E*zpe), vibrational entropy (TS_H), Gibbs free energies (*G*) and Gibbs free energy differences (Δ*G*) of Ti@(4 × 4)-Mo_3C_2-V_C at different adsorption sites.

	***E*/eV**	**E_{ZPE}/eV**	**TS_H/eV**	***G*/eV**	**Δ*G*/eV**
*+1/2H_2	-809.97	0.14	0.20	-810.03	0.00
*H(C^1)	-810.51	0.25	0.01	-810.27	-0.24
*H(C^2)	-810.33	0.25	0.01	-810.09	-0.06
*H(C^3)	-810.19	0.25	0.01	-809.95	0.08
*H(C^4)	-810.41	0.25	0.01	-810.17	-0.14
*H(C^5)	-810.27	0.25	0.01	-810.03	0.00
*H(Mo^1)	-810.62	0.18	0.01	-810.45	-0.42
*H(Mo^2)	-809.87	0.19	0.01	-809.69	0.34
*H(Mo^3)	-809.97	0.19	0.01	-809.79	0.24
*H(Ti^1)	-809.81	0.12	0.06	-809.75	0.28

Table S4. Calculated energies (*E*), zero-point energies (*E*zpe), vibrational entropy (TS_H), Gibbs free energies (*G*) and Gibbs free energy differences (Δ*G*) of V@(4 × 4)-Mo_3C_2-V_C at different adsorption sites.

	***E*/eV**	**E_{ZPE}/eV**	**TS_H/eV**	***G*/eV**	**Δ*G*/eV**
*+1/2H_2	-810.14	0.14	0.20	-810.20	0.00
*H(C^1)	-810.84	0.25	0.01	-810.60	-0.40
*H(C^2)	-810.55	0.25	0.01	-810.31	-0.11
*H(C^3)	-810.39	0.25	0.01	-810.15	0.05
*H(C^4)	-810.75	0.25	0.01	-810.51	-0.31
*H(C^5)	-810.47	0.25	0.01	-810.23	-0.03
*H(Mo^1)	-810.73	0.18	0.01	-810.56	-0.36
*H(Mo^2)	-810.04	0.19	0.01	-809.86	0.34
*H(Mo^3)	-810.15	0.19	0.01	-809.97	0.23
*H(V^1)	-810.15	0.14	0.04	-810.05	0.15

Table S5. Calculated energies (E), zero-point energies (Ezpe), vibrational entropy (TS_H), Gibbs free energies (G) and Gibbs free energy differences (ΔG) of Cr@(4 × 4)-Mo_3C_2-V_C at different adsorption sites.

	***E*/eV**	**E_{ZPE}/eV**	**TS_H/eV**	***G*/eV**	**ΔG/eV**
*+1/2H_2	-809.91	0.14	0.20	-809.97	0.00
*H(C^1)	-810.86	0.25	0.01	-810.62	-0.65
*H(C^2)	-810.82	0.24	0.01	-810.59	-0.62
*H(C^3)	-810.91	0.25	0.01	-810.67	-0.70
*H(C^4)	-810.94	0.25	0.01	-810.70	-0.73
*H(C^5)	-810.95	0.24	0.01	-810.72	-0.75
*H(Mo^1)	-811.22	0.19	0.01	-811.04	-1.07
*H(Mo^2)	-810.61	0.19	0.01	-810.43	-0.46
*H(Mo^3)	-810.61	0.19	0.01	-810.43	-0.46
*H(Cr^1)	-810.72	0.12	0.06	-810.66	-0.69

Table S6. Calculated energies (E), zero-point energies (Ezpe), vibrational entropy (TS_H), Gibbs free energies (G) and Gibbs free energy differences (ΔG) of Mn@(4 × 4)-Mo_3C_2-V_C at different adsorption sites.

	***E*/eV**	**E_{ZPE}/eV**	**TS_H/eV**	***G*/eV**	**ΔG/eV**
*+1/2H_2	-809.75	0.14	0.20	-809.81	0.00
*H(C^1)	-811.06	0.25	0.01	-810.82	-1.01
*H(C^2)	-811.02	0.24	0.01	-810.79	-0.98
*H(C^3)	-811.11	0.25	0.01	-810.87	-1.06
*H(C^4)	-811.14	0.25	0.01	-810.90	-1.09
*H(C^5)	-811.12	0.24	0.01	-810.89	-1.08
*H(Mo^1)	-811.37	0.19	0.01	-811.19	-1.38
*H(Mo^2)	-810.77	0.19	0.01	-810.59	-0.78
*H(Mo^3)	-810.77	0.19	0.01	-810.59	-0.78
*H(Mn^1)	-810.89	0.12	0.07	-810.84	-1.03

Table S7. Calculated energies (*E*), zero-point energies (*E*zpe), vibrational entropy (TS_H), Gibbs free energies (*G*) and Gibbs free energy differences (Δ*G*) of Fe@(4 × 4)-Mo_3C_2-V_C at different adsorption sites.

	***E*/eV**	**E_{ZPE}/eV**	**TS_H/eV**	***G*/eV**	**Δ*G*/eV**
*+1/2H_2	-809.34	0.14	0.20	-809.40	0.00
*H(C^1)	-810.19	0.25	0.01	-809.95	-0.55
*H(C^2)	-810.15	0.25	0.01	-809.91	-0.51
*H(C^3)	-810.20	0.25	0.01	-809.96	-0.56
*H(C^4)	-810.24	0.25	0.01	-810.00	-0.60
*H(C^5)	-810.25	0.25	0.01	-810.01	-0.61
*H(Mo^1)	-810.40	0.18	0.01	-810.23	-0.83
*H(Mo^2)	-809.86	0.19	0.01	-809.68	-0.28
*H(Mo^3)	-809.86	0.19	0.01	-809.68	-0.28
*H(Fe^1)	-810.05	0.14	0.04	-809.95	-0.55

Table S8. Calculated energies (*E*), zero-point energies (*E*zpe), vibrational entropy (TS_H), Gibbs free energies (*G*) and Gibbs free energy differences (Δ*G*) of Co@(4 × 4)-Mo_3C_2-V_C at different adsorption sites.

	***E*/eV**	**E_{ZPE}/eV**	**TS_H/eV**	***G*/eV**	**Δ*G*/eV**
*+1/2H_2	-808.73	0.14	0.20	-808.79	0.00
*H(C^1)	-808.85	0.25	0.01	-808.61	0.18
*H(C^2)	-808.88	0.25	0.01	-808.64	0.15
*H(C^3)	-808.95	0.25	0.01	-808.71	0.08
*H(C^4)	-808.97	0.25	0.01	-808.73	0.06
*H(C^5)	-808.98	0.25	0.01	-808.74	0.05
*H(Mo^1)	-809.10	0.18	0.01	-808.93	-0.14
*H(Mo^2)	-808.59	0.19	0.01	-808.41	0.38
*H(Mo^3)	-808.63	0.19	0.01	-808.45	0.34
*H(Co^1)	-808.86	0.15	0.04	-808.75	0.04

Table S9. Calculated energies (*E*), zero-point energies (*E*zpe), vibrational entropy (TS_H), Gibbs free energies (*G*) and Gibbs free energy differences (Δ*G*) of Ni@(4 × 4)-Mo_3C_2-V_C at different adsorption sites.

	***E*/eV**	**E_{ZPE}/eV**	**TS_H/eV**	***G*/eV**	**Δ*G*/eV**
*+1/2H_2	-807.85	0.14	0.20	-807.91	0.00
*H(C^1)	-807.94	0.25	0.01	-807.70	0.21
*H(C^2)	-807.94	0.25	0.01	-807.70	0.21
*H(C^3)	-808.05	0.25	0.01	-807.81	0.10
*H(C^4)	-808.07	0.25	0.01	-807.83	0.08
*H(C^5)	-808.09	0.24	0.01	-807.86	0.05
*H(Mo^1)	-808.20	0.18	0.01	-808.03	-0.12
*H(Mo^2)	-807.73	0.19	0.01	-807.55	0.36
*H(Mo^3)	-807.75	0.19	0.01	-807.57	0.34
*H(Ni^1)	-807.94	0.15	0.04	-807.83	0.08

Table S10. Calculated energies (*E*), zero-point energies (*E*zpe), vibrational entropy (TS_H), Gibbs free energies (*G*) and Gibbs free energy differences (Δ*G*) of Cu@(4 × 4)-Mo_3C_2-V_C at different adsorption sites.

	***E*/eV**	**E_{ZPE}/eV**	**TS_H/eV**	***G*/eV**	**Δ*G*/eV**
*+1/2H_2	-806.27	0.14	0.20	-806.33	0.00
*H(C^1)	-806.39	0.25	0.01	-806.15	0.18
*H(C^2)	-806.37	0.24	0.01	-806.14	0.19
*H(C^3)	-806.49	0.25	0.01	-806.25	0.08
*H(C^4)	-806.52	0.25	0.01	-806.28	0.05
*H(C^5)	-806.50	0.24	0.01	-806.27	0.06
*H(Mo^1)	-806.63	0.18	0.01	-806.46	-0.13
*H(Mo^2)	-806.18	0.19	0.01	-806.00	0.33
*H(Mo^3)	-806.18	0.19	0.01	-806.00	0.33
*H(Cu^1)	-806.25	0.14	0.06	-806.17	0.16

Table S11. Calculated energies (*E*), zero-point energies (*E*zpe), vibrational entropy (TS_H), Gibbs free energies (*G*) and Gibbs free energy differences (Δ*G*) of Ti@(4 × 4)-Mo_3C_2-$V_{surf\text{-}Mo}$ at different adsorption sites.

	***E*/eV**	**E_{ZPE}/eV**	**TS_H/eV**	***G*/eV**	**Δ*G*/eV**
*+1/2H_2	-810.00	0.14	0.20	-810.06	0.00
*H(C^1)	-810.20	0.24	0.01	-809.97	0.09
*H(C^2)	-810.30	0.25	0.01	-810.06	0.00
*H(C^3)	-810.34	0.25	0.01	-810.10	-0.04
*H(Mo^1)	-809.87	0.19	0.01	-809.69	0.37
*H(Mo^2)	-809.85	0.19	0.01	-809.67	0.39
*H(Mo^3)	-809.85	0.19	0.01	-809.67	0.39
*H(Mo^4)	-809.84	0.19	0.01	-809.66	0.40
*H(Mo^5)	-809.84	0.19	0.01	-809.66	0.40
*H(Ti^1)	-809.39	0.15	0.02	-809.26	0.80

Table S12. Calculated energies (*E*), zero-point energies (*E*zpe), vibrational entropy (TS_H), Gibbs free energies (*G*) and Gibbs free energy differences (Δ*G*) of V@(4 × 4)-Mo_3C_2-$V_{surf\text{-}Mo}$ at different adsorption sites.

	***E*/eV**	**E_{ZPE}/eV**	**TS_H/eV**	***G*/eV**	**Δ*G*/eV**
*+1/2H_2	-810.42	0.14	0.20	-810.48	0.00
*H(C^1)	-810.71	0.25	0.01	-810.47	0.01
*H(C^2)	-810.75	0.25	0.01	-810.51	-0.03
*H(C^3)	-810.74	0.25	0.01	-810.50	-0.02
*H(Mo^1)	-810.29	0.19	0.01	-810.11	0.37
*H(Mo^2)	-810.30	0.19	0.01	-810.12	0.36
*H(Mo^3)	-810.31	0.19	0.01	-810.13	0.35
*H(Mo^4)	-810.28	0.19	0.01	-810.10	0.38
*H(Mo^5)	-810.29	0.19	0.01	-810.11	0.37
*H(V^1)	-809.98	0.18	0.01	-809.81	0.67

Table S13. Calculated energies (E), zero-point energies (Ezpe), vibrational entropy (TS_H), Gibbs free energies (G) and Gibbs free energy differences (ΔG) of Cr@(4 × 4)-Mo_3C_2-$V_{surf\text{-}Mo}$ at different adsorption sites.

	E/eV	E_{ZPE}/eV	TS_H/eV	G/eV	ΔG/eV
*+1/2H_2	-809.74	0.14	0.20	-809.80	0.00
*H(C^1)	-810.08	0.25	0.01	-809.84	-0.04
*H(C^2)	-810.06	0.25	0.01	-809.82	-0.02
*H(C^3)	-810.05	0.25	0.01	-809.81	-0.01
*H(Mo^1)	-809.61	0.19	0.01	-809.43	0.37
*H(Mo^2)	-809.61	0.19	0.01	-809.43	0.37
*H(Mo^3)	-809.62	0.19	0.01	-809.44	0.36
*H(Mo^4)	-809.60	0.19	0.01	-809.42	0.38
*H(Mo^5)	-809.61	0.19	0.01	-809.43	0.37
*H(Cr^1)	-809.48	0.19	0.01	-809.30	0.50

Table S14. Calculated energies (E), zero-point energies (Ezpe), vibrational entropy (TS_H), Gibbs free energies (G) and Gibbs free energy differences (ΔG) of Mn@(4 × 4)-Mo_3C_2-$V_{surf\text{-}Mo}$ at different adsorption sites.

	E/eV	E_{ZPE}/eV	TS_H/eV	G/eV	ΔG/eV
*+1/2H_2	-809.11	0.14	0.20	-809.17	0.00
*H(C^1)	-809.47	0.25	0.01	-809.23	-0.06
*H(C^2)	-809.42	0.25	0.01	-809.18	-0.01
*H(C^3)	-809.43	0.25	0.01	-809.19	-0.02
*H(Mo^1)	-808.97	0.19	0.01	-808.79	0.38
*H(Mo^2)	-809.00	0.19	0.01	-808.82	0.35
*H(Mo^3)	-809.02	0.19	0.01	-808.84	0.33
*H(Mo^4)	-808.96	0.19	0.01	-808.78	0.39
*H(Mo^5)	-808.97	0.19	0.01	-808.79	0.38
*H(Mn^1)	-808.75	0.20	0.01	-808.56	0.61

Table S15. Calculated energies (E), zero-point energies (Ezpe), vibrational entropy (TS_H), Gibbs free energies (G) and Gibbs free energy differences (ΔG) of Fe@(4 × 4)-Mo_3C_2-$V_{surf\text{-}Mo}$ at different adsorption sites.

	***E*/eV**	**E_{ZPE}/eV**	**TS_H/eV**	***G*/eV**	**Δ*G*/eV**
$*+1/2H_2$	-807.90	0.14	0.20	-807.96	0.00
$*H(C^1)$	-808.30	0.25	0.01	-808.06	-0.10
$*H(C^2)$	-808.20	0.25	0.01	-807.96	-0.00
$*H(C^3)$	-808.20	0.25	0.01	-807.96	-0.00
$*H(Mo^1)$	-807.75	0.19	0.01	-807.57	0.39
$*H(Mo^2)$	-807.78	0.19	0.01	-807.60	0.36
$*H(Mo^3)$	-807.81	0.19	0.01	-807.63	0.33
$*H(Mo^4)$	-807.74	0.19	0.01	-807.56	0.40
$*H(Mo^5)$	-807.75	0.19	0.01	-807.57	0.39
$*H(Fe^1)$	-807.52	0.20	0.01	-807.33	0.63

Table S16. Calculated energies (E), zero-point energies (Ezpe), vibrational entropy (TS_H), Gibbs free energies (G) and Gibbs free energy differences (ΔG) of Co@(4 × 4)-Mo_3C_2-$V_{surf\text{-}Mo}$ at different adsorption sites.

	***E*/eV**	**E_{ZPE}/eV**	**TS_H/eV**	***G*/eV**	**Δ*G*/eV**
$*+1/2H_2$	-806.35	0.14	0.20	-806.41	0.00
$*H(C^1)$	-806.78	0.25	0.01	-806.54	-0.13
$*H(C^2)$	-806.66	0.25	0.01	-806.42	-0.01
$*H(C^3)$	-806.67	0.25	0.01	-806.43	-0.02
$*H(Mo^1)$	-806.19	0.19	0.01	-806.01	0.40
$*H(Mo^2)$	-806.24	0.19	0.01	-806.06	0.35
$*H(Mo^3)$	-806.28	0.19	0.01	-806.10	0.31
$*H(Mo^4)$	-806.20	0.19	0.01	-806.02	0.39
$*H(Mo^5)$	-806.21	0.19	0.01	-806.03	0.38
$*H(Co^1)$	-805.90	0.19	0.01	-805.72	0.69

Table S17. Calculated energies (E), zero-point energies (Ezpe), vibrational entropy (TS_H), Gibbs free energies (G) and Gibbs free energy differences (ΔG) of Ni@(4 × 4)-Mo_3C_2-$V_{surf\text{-}Mo}$ at different adsorption sites.

	E/eV	E_{ZPE}/eV	TS_H/eV	G/eV	ΔG/eV
*+1/2H_2	-804.64	0.14	0.20	-804.70	0.00
*H(C^1)	-805.01	0.25	0.01	-804.77	-0.07
*H(C^2)	-804.92	0.25	0.01	-804.68	0.02
*H(C^3)	-804.94	0.25	0.01	-804.70	0.00
*H(Mo^1)	-804.48	0.19	0.01	-804.30	0.40
*H(Mo^2)	-804.51	0.19	0.01	-804.33	0.37
*H(Mo^3)	-804.57	0.19	0.01	-804.39	0.31
*H(Mo^4)	-804.46	0.19	0.01	-804.28	0.42
*H(Mo^5)	-804.50	0.19	0.01	-804.32	0.38
*H(Ni^1)	-803.86	0.17	0.02	-803.71	0.99

Table S18. Calculated energies (E), zero-point energies (Ezpe), vibrational entropy (TS_H), Gibbs free energies (G) and Gibbs free energy differences (ΔG) of Cu@(4 × 4)-Mo_3C_2-$V_{surf\text{-}Mo}$ at different adsorption sites.

	E/eV	E_{ZPE}/eV	TS_H/eV	G/eV	ΔG/eV
*+1/2H_2	-802.06	0.14	0.20	-802.12	0.00
*H(C^1)	-802.43	0.25	0.01	-802.19	-0.07
*H(C^2)	-802.35	0.25	0.01	-802.11	0.01
*H(C^3)	-802.42	0.25	0.01	-802.18	-0.06
*H(Mo^1)	-801.94	0.19	0.01	-801.76	0.36
*H(Mo^2)	-801.93	0.19	0.01	-801.75	0.37
*H(Mo^3)	-801.97	0.19	0.01	-801.79	0.33
*H(Mo^4)	-801.89	0.19	0.01	-801.71	0.41
*H(Mo^5)	-801.91	0.19	0.01	-801.73	0.39
*H(Cu^1)	-800.99	0.15	0.03	-800.87	1.25
